# Practical Notes and Formulæ

**Published:** 1886-08-20

**Authors:** 


### PRACTICAL NOTES AND FORMULÆ.

**Blackberry Cordial.**—Take of—

| | | |
|---|---|---|
| ℞. | Blackberry juice | 2 pints, |
| | White sugar | 14 troy ozs, |
| | Powdered cloves | 2 drs, |
| | Powdered allspice | 2 drs, |
| | Powdered cinnamon | 2 drs, |
| | Powdered nutmeg | 2 drs, |
| | Brandy | 2 fl. ozs. |

—*Exchange.*

**Jamaica Dogwood.**—Dr. J. A. Mayes, in *American Practitioner and News*, writes about Jamaica dogwood and its value as an anodyne: I will state in one sentence my verdict—it has nearly all the good properties of opium, and none of the bad.

Its good effects are shown by relieving pain promptly, and by its soothing influence upon the nervous system, inducing sleep in almost every case, and being never followed by nausea or by the gastric disturbances that follow the use of opiates. * * *

Please try the following prescription for delirium tremens, or *mania a potu.* It has worked wonders in my hands:

Take of—

| | | |
|---|---|---|
| ℞. | Fluid extract erythroxylon cocoa | 6 drs, |
| | Fluid extract celery | 4 drs, |
| | Fluid extract Jamaica dogwood | 4 drs, |
| | Hoffman's anodyne, q. s. ad | 2 fl. ozs. |

Dose, 2 teaspoonfuls every two hours till sleep is induced; then 1 teaspoonful every three, four, or five hours till the nervous system returns to a normal condition.—*National Druggist.*

**French Shoe Polish.**—Take of—

| | | |
|---|---|---|
| ℞. | Vinegar | 2 pints, |
| | Soft water | 1 pint, |
| | Fine glue | 4 troy ozs, |
| | Logwood chips | 8 troy ozs, |
| | Powdered indigo | 2 drs, |
| | Bichromate of potassium | 4 drs, |
| | Tragacanth | 4 drs, |
| | Glycerin | 4 fl. ozs. |

Boil together, strain, and bottle.

**Menthol in the Treatment of Urticaria.**—*Union Medicale* for May 22, 1886, suggests the following simple remedy: Dissolve a small quantity of menthol in alcohol, and apply to the spots as a lotion. This preparation is said to be equally soothing in the case of insect-stings.

**A Paste for Use in Eczema.**—Funk (*Monatsch. f. prakt. Dermat.*, October, 1885) recommends the following:

| | | |
|---|---|---|
| ℞. | Salicylic acid | $2\frac{1}{2}$ drs, |
| | Ichthyol | 5 drs, |
| | Alcohol | 20 drs, |

To be rubbed in thoroughly with a brush twice daily.

**An Application to Prevent Scarring after Variola.**—Reimer (*St. Petersberger med. Woch.*, No. 20, 1885) speaks highly of the following salve, originally suggested by Schwimmer:

| | | |
|---|---|---|
| ℞. | Carbolic acid | 30 to 80 grs, |
| | Olive oil | 5 drs, |
| | Powdered chalk | 1 oz. |

Apply on a linen cloth, to be renewed twice daily. In thirteen hundred cases in which this ointment was used, deep scars were not once observed.—*N. Y. Medical Journal.*

**Elixir of Pepsin.**—The *National Druggist* gives the following formula for making elixir of pepsin from the scale pepsin.

| | | |
|---|---|---|
| ℞. | Pepsin, in scales | 128 grs, |
| | Lactic Acid | 30 min, |
| | Syrup | 6 fl. ozs, |
| | Alcohol | 4 fl. ozs, |
| | Compound elixir of taraxicum, No. 51 | 1 fl. oz, |
| | Water, q. s. ad | 16 ozs. |

Agitate the pepsin with 3 fluid ounces of water and the lactic acid until it is dissolved. Then add the syrup, compound elixir of taraxacum and the alcohol, and finally enough water to make 16 fluid ounces.

Each fluidram contains 1 grain scale pepsin.

**Meaning of Spiritus Vini Gallici.**—A correspondent of the *National Druggist* says: Please inform me what *spiritus vini Gallici* means, and whether in writing a prescription the physician should not designate what kind of brandy is meant, if French, California, or other?

Translated literally the above means the "spirit of French wine," the Latin adjective *gallicus* meaning Gallic or French. Originally, perhaps, all brandies were of French origin, whence the name, but that must have been a long time ago. Now the term, according to United States Pharmacopœa, means "an alcoholic liquid obtained by the distillation of fermented grapes, and at least four years old."

**Glycerin in Inhalation for Cough.**—Dr. Trastour, of Nantes, has been very successful in treating obstinate cough with glycerin vapor. Five or 6 drops of this liquid are placed in a china capsule and heated over a spirit lamp; a quantity of vapor is thus given off, the inhalation of which gives great relief. This remedy has been found particularly useful to phthisical patients.—*American Practitiouer and News.*

